# Justifying Our Credences in the Trustworthiness of AI Systems: A Reliabilistic Approach

**DOI:** 10.1007/s11948-024-00522-z

**Published:** 2024-11-21

**Authors:** Andrea Ferrario

**Affiliations:** 1 Institute of Biomedical Ethics and History of Medicine, University of Zurich, Zurich, Switzerland; 2 ETH Zurich, Zurich, Switzerland

**Keywords:** Artificial intelligence, Trustworthiness, Trustworthy AI, Epistemology, Justification, Reliabilism, Belief, Credence

## Abstract

We address an open problem in the philosophy of artificial intelligence (AI): how to justify the epistemic attitudes we have towards the trustworthiness of AI systems. The problem is important, as providing reasons to believe that AI systems are worthy of trust is key to appropriately rely on these systems in human-AI interactions. In our approach, we consider the trustworthiness of an AI as a time-relative, composite property of the system with two distinct facets. One is the actual trustworthiness of the AI and the other is the perceived trustworthiness of the system as assessed by its users while interacting with it. We show that credences, namely, beliefs we hold with a degree of confidence, are the appropriate attitude for capturing the facets of the trustworthiness of an AI over time. Then, we introduce a reliabilistic account providing justification to the credences in the trustworthiness of AI, which we derive from Tang’s probabilistic theory of justified credence. Our account stipulates that a credence in the trustworthiness of an AI system is justified if and only if it is caused by an assessment process that tends to result in a high proportion of credences for which the actual and perceived trustworthiness of the AI are calibrated. This approach informs research on the ethics of AI and human-AI interactions by providing actionable recommendations on how to measure the reliability of the process through which users perceive the trustworthiness of the system, investigating its calibration to the actual levels of trustworthiness of the AI as well as users’ appropriate reliance on the system.

## Introduction

Let us consider the following scenario:

Scenario(Belief): Let *X* be a human agent. At a certain time, *X* believes that ***P***, where ***P*** = “The AI system *S* is trustworthy.”

For instance, *X* could be a physician who may believe that a medical AI that predicts the onset of sepsis in intensive care patients (Fleuren et al., 2020) is ‘trustworthy’ and acts on their belief accordingly. Alternatively, *X* could be a web designer interacting with an AI-infused app that generates images from textual prompts. *X* recommends the app to peers as they certified its ‘trustworthiness’ over time (Bommasani et al., 2021). Clearly, many other examples can be introduced, which cover virtually all instances where humans interact with AI systems in modern applications. In this paper, motivated by the cases offered by Scenario(Belief), we address the following question:

What does it make an epistemic stance, such as belief, on the trustworthiness of an AI *justified*?

Questions on the justification of our epistemic stances toward the trustworthiness of AI are important for the philosophy of AI, and, in particular, the ethics of AI. In general, we would like to think that a physician holds a well-grounded belief on the trustworthiness of an AI system before using its predictions for their clinical decision-making. Namely, their acting upon these predictions should follow a justified stance on whether the AI is worthy of their trust or not. From an epistemological perspective, justifications would separate well-grounded stances from those that may have been the result of fortuitous processes (‘epistemic luck’). In fact, justified stances on the trustworthiness of AI are seen as a necessary step to appropriately relying on these systems (Durán & Jongsma, 2021). Ethically, deploying trustworthy AI and holding well-grounded stances on them may mitigate the risk of unethical decisions affecting the lives of many people (Jobin et al., 2019). In particular, without giving reasons to believe that an AI is worthy of trust, we cannot ascribe moral responsibility in human-AI interactions appropriately (Durán & Jongsma, 2021), as well as efficiently mitigating the risks arising from the erosion of the epistemic authority of professionals (Grote & Berens, 2020). The design and development of AI that are worthy of our trust can ensure that principles such as fairness, transparency, and accountability are upheld (European Commission, 2018). By fostering trustworthiness, AI systems can adhere to standards that protect user rights and maintain public confidence, thereby enabling the responsible and sustainable integration of these technologies into society. In addition, an account of ‘justificatory AI’ that could emerge from answering our question has the potential to inform research on human-AI interactions, supporting the ongoing debate on how to entrench trust—whatever that may mean—in the trustworthiness of AI (Lee & See, 2004; Jacovi et al., 2021; Ferrario & Loi, 2022) and foster appropriate reliance in these systems in empirical studies (Schemmer, 2022; Schlicker et al., 2022).

In this work, our answer to the above question is a reliabilistic account of justification of the epistemic attitudes called credences–informally: ‘beliefs with degrees’–that appropriately describe the stance towards the trustworthiness of AI resulting from the interaction with these systems. Our approach comprises the following, three steps.

First, we discuss what is *X*’s belief in Scenario(Belief) about. In fact, the definition and characterization of the trustworthiness of AI systems is still an open problem in the philosophy of AI (Floridi, 2019). Most researchers, private organizations, and institutions promote the idea that trustworthiness is a *composite* property of AI systems that descends from ethical principles governing the design and development of AI in our society (European Commission, 2018; Floridi, 2019; Jacovi et al., 2021). This ‘Trustworthy AI’ paradigm then focuses on the properties constituting what would make AI worthy of users’ trust–e.g., being accurate, technically reliable, and transparent. Inspired by the literature on ‘contractual trust’ in AI (Jacovi et al., 2021; Hawley, 2014), we elaborate on the ‘Trustworthy AI’ position by taking a context- and time-relative perspective on the trustworthiness of AI systems. Doing so, we do not aim to describe what properties trustworthiness should entail in every possible application, but, rather, to clarify how trustworthiness manifests and relates itself with the AI’s users throughout the life cycle of an AI system in a given context. We show that at each step of the AI system’s life cycle and moment of time, its trustworthiness comprises two, measurable facets. The first describes the objective level of trustworthiness of the system. The second provides a point-in-time perspective on how a deployed system is subjectively perceived as being worthy of trust by its users.

Then, we show that a probabilistic account of vindicated credences (Tang, 2016) allows encoding the different facets of the trustworthiness of AI systems. In particular, their vindication aligns the degree of confidence of the credence, i.e., the level of perceived trustworthiness, to the actual trustworthiness of an AI system *via* the system’s objective probability to be worthy of trust. Thus, vindication provides a definition of what we call ‘calibration’ between the actual and perceived trustworthiness of an AI system.

Finally, we introduce our reliabilistic account of justified credence in the actual trustworthiness of AI systems. It is derived from Tang’s ‘(Probability)’ reliabilistic theory of justified credence (Tang, 2016). Our account states that a credence in the trustworthiness of an AI system is justified if and only if it is the result of a reliable assessment process, i.e., a process to assess the trustworthiness of AI that tends to result in a high proportion of vindicated credences.

In summary, our work contributes to the philosophy of AI providing an account of justification of the credences we hold on the trustworthiness of AI systems. In particular, it contributes to the ethics of AI by introducing a measurable definition of the calibration between the perceived and actual trustworthiness of an AI system, as well as a measure of the reliability of the process of assessing its trustworthiness. These results support our understanding of appropriate forms of trust in AI, specifically, trust relations that are grounded in the systems’ trustworthiness (Ferrario & Loi, 2022). Finally, it informs empirical research in human-AI interactions by suggesting the design of studies to investigate the relation between the reliability of the assessment process, the different manipulations of the trustworthiness of the AI over time, the provision of methodologies to support users’ assessments, and the appropriate reliance on the AI’s predictions (Schemmer, 2022).

## The Trustworthiness of AI Systems

### What Makes AI Worthy of Trust?

In interpersonal relations, trustworthiness is the property of an individual that, according to the Stanford Encyclopedia of Philosophy (SEP), “is $\dots$ a kind of reliability, although it’s not obvious what kind” (McLeod, 2021). As the quote from the SEP suggests, it is not easy to encode what makes someone worthy of trust. A similar difficulty affects the discourse on the trustworthiness of AI, which aims to characterize what makes an AI system worthy of our trust (Kaur et al., 2022). Addressing this issue is relevant, as defining the trustworthiness of AI helps ensuring that these systems are aligned with ethical principles and adhere to social norms. In fact, the idea is that a trustworthy AI system is designed in such a way that ethical, social, economic, and legal concerns are appropriately addressed. This achievement would foster broader public trust in AI and facilitate its adoption across various sectors. Thus, over time, researchers and their organizations, private companies, and government agencies have attempted to describe what a trustworthy AI system should entail and provide methods to assess the trustworthiness of AI. These efforts, collectively named ‘Trustworthy AI,’ have produced an extensive literature discussing *principled* suggestions of which properties^1^ make AI systems worthy of our trust.^2^ For instance, the European Commission’s (EU) High-Level Expert Group ‘Ethics guidelines for trustworthy AI’ states that trustworthy AI systems should be lawful, ethical, and robust both from a technical and social perspective, promoting principles, including the respect of human agency and oversight, technical robustness, safety, and transparency (European Commission, 2018). These principles determine a series of properties that a trustworthy AI should possess, such as being accurate, resilient to attacks, fair, technically reliable, reproducible, and explainable (Floridi, 2019; Li et al., 2023; Mattioli et al., 2024). Underlying the ‘Trustworthy AI’ paradigm is the idea that principles suggest the properties that constitute the trustworthiness of an AI system (Laux et al., 2024). However, principles alone are not sufficient to fully guide the development of trustworthy AI systems. In fact, the importance of these properties is contextual, varying based on the designers’ intentions, the system’s function, and its specific application (Novelli et al., 2023). For instance, the significance of transparency or accuracy in AI systems used for healthcare or automated driving differs from that in AI systems classifying emails as spam or supporting personalized marketing. Similarly, the reliability of an AI system predicting critical industrial machinery failures is more crucial than the reliability of generative AI systems creating digital art–where lower reliability might even enhance creativity. The necessary contextualization of these principle-based properties is provided by the now influential ‘contractual trust’ approach in human-AI interactions (Jacovi et al., 2021). Drawing on Hawley’s work on trust with commitments (Hawley, 2014), this approach describes AI trustworthiness in terms of ‘contracts,’ namely, explicit, predetermined properties that an AI must maintain during use, as deemed relevant to its application context. These properties are typically selected and implemented by the system’s designers based on the intended future application of the AI. Then, an AI “is trustworthy to some contract if it is capable of maintaining this contract” (Jacovi et al., 2021, p.  627).

Altogether, the ‘Trustworthy AI’ paradigm and ‘contractual trust’ approach suggest that what makes an AI system worthy of our trust in a given context is its capability to function in such a way that is aligned with a set of predetermined expectations for that system in that context. These expectations, in turn, are aligned with ethical principles that govern the design and development of AI in a social system. Paraphrasing Forrest Gump: “Worthy of trust is as trustworthy *does*.” Importantly, trustworthiness has to be assessed. Different guidelines for trustworthy AI explicitly mention the need to assess the properties constituting the system’s trustworthiness by quantitative and qualitative methods altogether (ISO24028:2020, 2020; Kaur et al., 2022). For instance, technical robustness and safety can be assessed by penetration testing, accuracy by measuring performance metrics, and transparency by different types of fact sheets (Sokol & Flach, 2020). In general, qualitative methods can be followed by scoring rules to generate numerical scores, if required.

A few final comments. Some authors argue that AI systems cannot be worthy of trust as they lack human-like capacities such as mental states, or the ability to be held accountable for their actions (Ryan, 2020). This view stems from the direct transfer of expectations about trust and trustworthiness from interpersonal relations to human-AI interactions. The main limitation of this approach is that it tries applying multifaceted and contested concepts, like trust and trustworthiness in interpersonal relations, to a fundamentally different context–i.e., human-AI interactions–without adequately justifying the appropriateness of this manoeuvre. After all, it is just one possible approach to this topic and should be better defended against alternatives. The ‘Trustworthy AI’ paradigm, the contractual trust approach introduced above, or even motive-agnostic accounts of trust (Taddeo, 2010; Loi et al., 2023), appear to be more suitable frameworks for characterizing AI trustworthiness. These perspectives entrench the trustworthiness of AI in the technical capabilities of this technology, while also integrating the human factors–such as fears and expectations AI induces at the individual, organizational, or societal level–into ethical principles guiding these properties, and the contracts the AI is required to uphold among others. In doing so, they avoid the criticisms typically associated with affective, normative, and risk-based accounts of trust and their implications for trustworthiness, while focusing on the practical aspects of AI ethics and human-AI interaction research. Finally, we note that ‘Trustworthy AI’ is investigated also in the human-AI interaction domain, where research promotes the idea that, manipulating *the levels* of trustworthiness of an AI and collecting the self-reported measures of trust of its users it is possible to assess empirically whether ‘trust is calibrated with trustworthiness’ (Lee & See, 2004; Jacovi et al., 2021). Here, the goal is to inform the design of AI systems that are worthy of trust manipulating the context-relative and measurable properties that comprise their trustworthiness. Calibrating trust in the trustworthiness of AI systems is ethically desirable, as it fosters trust grounded in systems that perform as expected. This approach safeguards user autonomy and prevents deception or misuse of technology, thereby promoting responsible AI deployment and enhancing user confidence in AI applications (Lee & See, 2004; Jacovi et al., 2021). However, understanding what makes an AI worthy of trust and the analysis of its relation with the mental processes and actions of its users–including trust–requires more than the general characterization of trustworthiness we have provided so far. We discuss this point in the next section.

### Objective and Subjective Dimensions of Trustworthiness

In line with recent, empirical literature on AI (Liao & Sundar, 2022; Schlicker et al., 2022; Zerilli et al., 2022), we distinguish between the objective and subjective facets of trustworthiness that arise in human-AI interactions. To do so, it is useful to introduce a time-relative perspective on AI, representing the high-level life cycle of an AI as a sequence of alternating design and deployment phases, and discussing trustworthiness in relation to the different steps of the system’s life cycle.^3^ During the design process of an AI, designers identify the properties constituting the trustworthiness of the to-be AI system, given its intended use and application and introduce the measurability criteria for each property. After design, the AI is deployed and starts functioning (i.e., computing predictions). However, the environment in which the AI is deployed changes over time as customers’ interests may exhibit seasonality, the stock market is likely to fluctuate, and new drugs may be used in clinical practice. Therefore, data distributions change—a phenomenon known as concept shift (Žliobaitė et al., 2016). Concept shift compromises, for instance, the accuracy and fairness of the AI (Floridi et al., 2022). It can invalidate explanations of AI predictions, while new possibilities to violate the safety measures of the system arise over time.

These considerations suggest that the properties that contribute to the trustworthiness of an AI are time-relative. For each property that may contribute to the trustworthiness of an AI as specified in a set of contracts for that system, e.g., accuracy and fairness, we argue there exists a true (yet unknown) level that degrades over time and that is approximated by designers’ estimates, which, in turn, are based on the empirical data available at system’s (re-)design.^4^ With this in mind, we can consider the trustworthiness of an AI system *S* as a second-order property of the system that is constituted by a finite number of time-relative, and measurable system’s properties. These, in turn, are specified by a set of contracts that *S* is required to maintain in an application context. In particular, the *actual* level of trustworthiness of system *S* at time *t*–notation: $\texttt {Tw}_S(t)$–depends on the property levels at that time.

Let us discuss our perspective on trustworthiness in more detail. First, we note that the ‘derived’ nature of the trustworthiness of AI, namely, that trustworthiness is a second-order property of an AI system, is not a novelty suggested exclusively by the ‘Trustworthy AI’ paradigm or the contractual trust account. Back in time, in their 2004 seminal work on trust in automation, when describing ‘trust calibrated in trustworthiness,’ Lee and See define calibration as “the correspondence between a person’s trust in the automation and the automation’s capabilities” (Lee & See, 2004, p. 55). These capabilities (plural) constitute the trustworthiness of the system. More recently, Mattioli and colleagues state that: “A trustworthy software is defined [...] by a *combination* of overlapping properties: reliability, safety, security, privacy, availability and usability. For a [machine learning]-based system, this translates and extends to accuracy, robustness, fairness, accountability, transparency, explainability and ethics” (Mattioli et al., 2024, p. 17, emphasis is ours). The idea that trustworthiness is a cumulative (or aggregate) property of a possibly artificial agent is promoted also in the case of information sources (Kaur et al., 2021; Primiero et al., 2023). Further, by the our definition, the trustworthiness of an AI is a context- and time-relative as well as measurable property of the system. $\texttt {Tw}_S(t)$ is the unknown, true level of trustworthiness of the system *S* at time *t*. We call it the level of ‘actual trustworthiness’ of *S* at time *t*. Our approach suggests that $\texttt {Tw}_S(t)$ results from the aggregation of the (unknown) levels at time *t* of all the properties that constitute trustworthiness.^5^ During the design of the AI, i.e., at time $t_0$, these levels are approximated by the designers’ estimate $\widehat{\texttt {Tw}}_S(t_0)$, which is typically obtained through extensive testing. After deployment, designers can mitigate the effect of time on the actual trustworthiness of the system through the redesign of the AI.

However, when discussing the trustworthiness of an AI we need to consider a second, subjective facet. In fact, after its deployment, users interact with the AI and its assessment process takes place. To assess the trustworthiness of an AI is to interact with the system and assess its degree of trustworthiness with requirements that are relevant for its context and users. Here, with the term ‘assessment’ we mean “the action or an instance of making a judgment about something.”^6^ The judgement can also be formal, possibly a quantification of the system’s trustworthiness. It depends on the type of user and the goal of their interaction with the AI system.^7^ For instance, a physician assesses the trustworthiness of a system classifying medical images each time he needs to rely on the AI to identify a bone fracture. The assessment is performed thoroughly before using the AI prediction for decision-making. It comprises a visual check of the medical image, access to additional medical information, such as an explanation of the prediction, similar images with that type of bone fracture, or annotations by other healthcare professionals. Then, the assessment process ${\mathcal {A}}_S$ of the AI system *S* can be defined as the subset of the interaction process comprising all sequences of user-*S* interactions whose steps contain subjective judgments of the pre-determined, trustworthiness-relevant properties of *S*. For instance, checking the prediction of a medical AI would enable the assessment of its explainability—if explainability is considered as contributing to the system trustworthiness (Durán & Jongsma, 2021; Ferrario & Loi, 2022). Modifying user credentials or import data may support the assessment of the safety, or technical reliability of the AI instead.

Crucially, the procedures a user can apply to assess the trustworthiness of an AI during the assessment process are different from those performed during the design process of the system itself. In fact, a user does not typically have the capability to run thousands of penetration tests to measure the safety of the system under consideration at the moment of decision-making. Nor could a user assess the fairness of the AI by accessing the dataset used for training and testing the implementation of the chosen measure before choosing to rely on the system or not. Similar considerations hold for other properties of the system trustworthiness, such as accuracy, and robustness. Instead, what users may leverage to assess the trustworthiness of the system and support their decision-making are ‘certifications’ of trustworthiness and its properties by third parties, such as auditing and regulatory bodies (Floridi et al., 2022). A recent example are the ‘certification labels for trustworthy AI’ (Scharowski et al., 2023), which reflect a credible auditing process and provide users with cognitively-accessible and effective trustworthiness cues. Further, users may also leverage social transparency cues, such as the summaries of the trustworthiness assessments performed by other users (Ehsan et al., 2021; Knowles & Richards, 2021). These methods are crucial for helping users assess the trustworthiness of an AI system, which is initially defined and implemented through ascribed properties, or ‘contracts,’ by its designers. By enhancing the transparency of these properties, we can reduce the risk of misalignments between designers and users regarding what makes an AI system worthy of trust. The result of the assessment process (instance) performed by user *X* at time *t* is the perceived trustworthiness of the system by that user at that time. We denote $\widehat{\texttt {PTw}}_S(t,X)$ as the level of perceived trustworthiness of system *S* at time *t* by *X*. Without any loss of generality, we assume that $\widehat{\texttt {PTw}}_S(t,X)\in [0,1]$ for all users *X* and time *t*.^8^ We also posit that, in general, $\texttt {Tw}_S(t) \ne \widehat{\texttt {PTw}}_S(t,X)$, for all $t{>}t_0$. In fact, depending on how they interact with the ‘trustworthiness cues’ of the system (Liao & Sundar, 2022), users may over- or underestimate the level of its actual trustworthiness.

## Believing with Degrees in the Trustworthiness of AI

### Credences and their Vindication

Let us turn our attention to the epistemic attitudes on the trustworthiness of AI systems with a remark: beliefs admit of truth or falsity. In fact, we either believe that *p* or not (including withholding it), and when we do, we believe *p* is true. However, sometimes we do not believe that *p*, but, rather, we have a certain *degree of confidence* in it. E.g., we may believe we are sitting in a chair at a certain moment of time, but we may just be somewhat confident that tomorrow it will not snow, or that our friends are being honest with us. The propositional attitude called ‘credence’ formalizes these uncertain, everyday stances. More formally, a credence of *x* in *p* is the attitude that expresses our degree of confidence, i.e., *x*, that *p* (Eriksson & Hájek, 2007; Jackson, 2020). The degree of confidence *x* lies in the interval between 0 and 1 inclusive; the value 0 resp. 1 represents maximal confidence that *p* is false resp. true (Jackson, 2020). We denote *X*’s credence of *x* in *p* at time *t* as $c_{(t,X)}[p]=x$. (Keeping the time and agent subscripts will be useful when discussing credences in ${\varvec{P}}$, where as in ${\varvec{P}}$ Scenario(Belief).

Multiple authors share the idea that, differently from beliefs, credences are not either true or false (Hájek, 2011; Carr, 2015; Tang, 2016, 2021).^9^ Rather, they are in relation with other notions of ‘correctness,’ such as objective probability (Tang, 2016, 2021).^10^ For instance, a credence of 0.5 that tomorrow will rain seems to be ‘correct’ if the objective probability that tomorrow it will rain is equal to 0.5. To this end, Háyek’s proposes the motto “[t]ruth is to belief, as agreement to objective chance is to degree of belief” (i.e., a credence) (Hájek, 2011, p. 2). Then, when talking about credences we assume that *p* is endowed with an objective probability of being true that the degree of confidence of our credence may approximate. (For the moment, we leave aside the problem of interpreting this probability.) The following is an important definition:

#### Definition 2.1

(**Vindicated credence** (Van Fraassen, 1983; Pettigrew, 2013; Tang, 2016)) A credence of *x* in *p* is *vindicated* if and only if the objective probability of *p* being true is *x*.

Here, we suggest to consider credences in relation to the trustworthiness of an AI system instead of binary beliefs. That is, we propose to replace Scenario(Belief) with

Scenario(Credence): Let *X* be a human agent. At a certain time, *X* holds a credence of *x* in ***P***, where ***P*** = “The AI system *S* is trustworthy.”

The reasons for this are as follows. First, credences intercept the different stances that human agents may have towards the trustworthiness of AI systems. In fact, real-world human-AI interactions suggest to assume that these stances have degrees of confidence and that these degrees depend on contextual- and time-relative factors. We can show this point considering the example of a physician who interacts with a medical AI that classifies medical images of bone fractures. The physician may be highly confident that the AI is technically reliable because he never suffered a service interruption in months. At the same time, he may have very little confidence that the system is accurate due to recent cases of mislabeling medical images. Overall, the physician’s perceived level of trustworthiness is a function of his degree of confidence in the properties (e.g., technical reliability and accuracy) that constitute the system’s trustworthiness. In fact, the trustworthiness of AI systems comprises different properties in which we generally hold credences with different degrees of confidence. In particular, the level of perceived trustworthiness is the degree of confidence of the physician’s credence in the trustworthiness of the AI (at that given time).

Further, endorsing a probabilistic account of vindicated credences in ${\varvec{P}}$ we can capture the two facets of the trustworthiness of an AI system we discussed in Section “The Trustworthiness of AI Systems”. In fact, ${\varvec{P}}$ refers to a property, namely, the actual trustworthiness of the AI, which, in turn, is assessed by its users over time. We are in a situation analogous to this example by Háyek:

“And much as one’s subjective estimate of the mass of an object ‘aligns with the truth’ to the extent that it approximates the objective mass of that object, so one’s subjective probability of an event ‘aligns with the truth’ to the extent that it approximates the objective probability of that event” (Hájek, 2011,  p. 13).

We will further elaborate on this point in the following section.

### Credences in ${\varvec{P}}$, Their Objective Probabilities, and the Facets of Trustworthiness

To continue investigating credences in ${\varvec{P}}$, we consider the following steps: (1) to clarify the role of the perceived trustworthiness of an AI in an account of credences in ${\varvec{P}}$, such as in Scenario(Credence), (2) to characterize the objective probability of the credences in ${\varvec{P}}$, and (3) to discuss the role of vindication of these credences.

Let us discuss (1) by considering a user *X* who holds a credence in ${\varvec{P}}$ at time *t* for a system *S* as the result of assessing its trustworthiness. As remarked in our example above, it seems natural to state that the level of perceived trustworthiness $\widehat{\texttt {PTw}}_S(t,X)$ is *X*’s subjective degree of confidence that the system *S* is trustworthy at time *t*: in fact, the higher the level $\widehat{\texttt {PTw}}_S(t,X)$, the more confident *X* is that the AI is worthy of trust at time *t*. (By definition, $\widehat{\texttt {PTw}}_S(t,X)\in [0,1]$.) In other words, $c_{(t,X)}[{\varvec{P}}]=\widehat{\texttt {PTw}}_S(t,X)$.

Step (2) requires to clarify what the objective probability that ${\varvec{P}}$ at time *t* for *S*—in symbols: $r^{{\varvec{P}}}_S(t)$—entails. Here, we suggest to take a ‘technical artifact’ perspective on AI systems, namely, to consider them as the result of human agents’ intentionality and the production capabilities of technology (Borgo et al., 2014). To do so, we start by considering each AI system *S* as an individual of an ‘AI kind’ that we still denote by *S* with a slight abuse of notation. For instance, a company could design *N* copies of a medical AI system and deploy them in different healthcare centers, or an AI system classifying documents used in certain companies and deploy them across their branches. We refrain from an in-depth discussion about the possible ontology of AI systems and a precise definition of their ontological kinds. Here, we simply assume that there exists a meaningful way to group AI systems in ‘kinds,’ which can be *prima facie* characterized by the intended function that all the individual artifacts in that kind are designed to execute.^11^ This is sufficient for clarifying step (2). Then, following Háyek’s motto on truth for credences (Hájek, 2011) and Longy’s account of the objective probability for artefacts to be ‘well-functioning’ (Longy, 2006), we interpret $r^{{\varvec{P}}}_S(t)$ as the objective chance that individual AI systems of the kind *S* execute their intended function *correctly* at time *t*. Here, ‘correctly’ refers to the properties that are ascribed at design to all systems of the kind *S* under consideration and are supposed to be maintained by these systems after their deployment. Therefore, following our discussions from Section “The Trustworthiness of AI Systems”, it is the trustworthiness of these systems that encodes these properties, which, in turn, are expression of pre-determined contracts. Given those premises, we interpret the objective probabilities that the systems in the kind *S* work correctly as follows. Let us suppose that *N* copies of a system *S* are designed at time $t_0$ and successfully deployed in their respective environments. By design, all copies $S_i$ start with the same level of actual trustworthiness $\texttt {Tw}_{S}(t_0)$. Then, let us suppose there exists a threshold $a_S{>}0$ that discriminates between trustworthy and not trustworthy systems in the kind *S*. The threshold may be specified by designers to indicate the minimum level of actual trustworthiness that each system $S_i$ should satisfy over time to be considered still worthy of trust, i.e., a system that functions correctly. Then, $a_S$ is a quantification of a quality standard of the systems $S_i$, i.e., their actual trustworthiness, that is specified during their design and it is valid throughout their lifetime. (For simplicity, we assume that the threshold is constant over time. Also, it seems natural that different kinds of AI systems would be characterized by different thresholds.) At each time $t{>}t_0$, let us suppose to perform *N* independent experiments to ascertain whether each system $S_i$ is trustworthy or not using $a_S$ as a reference threshold for its levels of actual trustworthiness $\texttt {Tw}_{S_i}(t)$. Each experiment is a Bernoulli trial. (The output of each experiment is binary: the system is trustworthy or not.) Then, the objective probability $r^{{\varvec{P}}}_{S_i}(t)$ of the system $S_i$ to be trustworthy at time *t* is interpreted as the probability of the *i*-th Bernoulli trial in the experiment at time *t*. The objective probabilities $r^{{\varvec{P}}}_S(t)$ encode and quantify the stochasticity of the event ${\varvec{P}}$. In general, they are unknown quantities, as is customary for the probabilities of a multitude of stochastic processes with binary outcomes in domains such as physics (e.g., radioactive decay), biology (e.g., gene expression), medicine (e.g., development of sepsis in a patient admitted to the intensive care unit), industry (e.g., predictive maintenance of machines), and many others.^12^ Nowadays, researchers and data scientists typically model these probabilities using context-specific empirical evidence and machine learning algorithms, including those of deep learning. In our setting, the utility of introducing the objective probabilities $r^{{\varvec{P}}}_S(t)$ is primarily theoretical: they enable formalizing the justification of credences in the trustworthiness of AI systems, as we started discussing in this section and will show in detail in what follows. For practical purposes, such as empirical studies investigating how AI users hold credences in the trustworthiness of a system, researchers can model these probabilities using contextual information that encodes the design and deployment history of the corresponding system–e.g., the number of past retrainings or critical malfunctions and the measurements of the past levels of its actual trustworthiness. This modeling approach is expressed by Longy as follows: “the [objective] probability an artefact item has to be a well-functioning one depends on the conditions and processes of its manufacture, and these, in turn, depend for their maintenance or improvement on a great many factors” (Longy, 2006, p.94). Then, it turns out that our interpretation of $r^{{\varvec{P}}}_S(t)$ as objective chance is a time-relative version of Longy’s account for the objective probability of artefacts to be well-functioning.^13^

Finally, on step (3): the vindication of the credences in ${\varvec{P}}$—see def. 2.1—allows relating actual to perceived levels of trustworthiness of the system *S*. In fact, from def. 2.1, it follows that *X*’s credence of ${\widehat{\texttt {PTw}}}_S(t,X)$ in the trustworthiness of the AI system *S* at time *t* is vindicated if and only if

1 $$\begin{aligned} c_{(t,X)}[{\varvec{P}}]= {\widehat{\texttt {PTw}}}_S(t,X) = r^{{\varvec{P}}}_S(t). \end{aligned}$$

Equation (1) states that for a credence in the trustworthiness of an AI system at time *t* to be vindicated, the perception of the actual level of trustworthiness of the AI is equal to the system’s objective probability to be trustworthy at that moment. As previously discussed, this probability is a function of the past levels of actual trustworthiness of the system, among others. Therefore, endorsing a probabilistic account of credence in the trustworthiness of an AI (instead of beliefs) allows relating the objective and subjective facets of the system’s trustworthiness via the vindication of such credences.^14^ (In what follows, we will remove the adjective ‘actual’ when discussing credence in the trustworthiness of an AI whenever possible for the sake of readability.)

Lastly, we note that eq. (1) provides a natural definition of the calibration of the perceived levels of the trustworthiness of an AI over time. In fact, we can state that *X*’s level of perceived trustworthiness $\widehat{\texttt {PTw}}_S(t,X)$ is calibrated at time *t* to the actual trustworthiness of the AI system *S* if and only if *X*’s credence of $\widehat{\texttt {PTw}}_S(t,X)$ in the actual trustworthiness of *S* at time *t* is vindicated. Then, a user’s perception of the trustworthiness of an AI is calibrated to the actual level of trustworthiness of the system if the degree of confidence, i.e., the level of *perceived* trustworthiness, of the user’s credence in the *actual* trustworthiness of the system reflects the *objective probability* that the system is trustworthy at that given time. A user’s calibrated level of perceived trustworthiness is low, resp. high, when the objective probability of the AI being trustworthy is low, resp. high, at that time. Therefore, calibration allows expressing when a level of perceived trustworthiness is the result of an assessment process (token) through which a user was able to hold a ‘correct’ credence in the actual trustworthiness of the AI. This correctness relates the perception of the actual facet of trustworthiness of the system *S* to the objective probability $r^{{\varvec{P}}}_S(t)$ as described in eq. (1). This, we argue, provides a simpler and more precise definition of the calibration process than what is commonly promoted by the ‘trust calibrated in trustworthiness’ approach, which, in turn, requires defining trusting, introducing a quantification of its levels, and relating them to the different facets of trustworthiness of an AI system in a causal way (Lee & See, 2004; Jacovi et al., 2021).

It remains to discuss how to justify credences in the trustworthiness of an AI and whether calibration between perceived and actual trustworthiness can be measured quantitatively and analyzed in studies on human-AI interactions. We tackle these topics in the forthcoming section.

## Justifying Credence in the Trustworthiness of AI Systems: A Reliabilistic Account

Similarly to the case of a true belief that is the result of an accidental, coincidental, or fortuitous belief-forming process, a credence may be validated but not justified. Therefore, we now introduce our reliabilistic account of justified credence in the trustworthiness of an AI. It derives from Tang’s reliability theory of justified credence named ‘(Probability)’ (Tang, 2016), applying it to the case of a probabilistic account of credence in the trustworthiness of AI systems. It is reliabilistic in the sense that we ascribe reliability to the process assessing the trustworthiness of an AI.

Tang’s ‘(Probability)’ account states that the assessment process ${\mathcal {A}}_S$ of an AI system *S* is reliable if it tends to result in a high proportion of credences that match or approximate the corresponding objective probabilities of the system *S* being trustworthy (Tang, 2016). By definition, in Tang’s ‘(Probability)’ account, the assessment process is reliable if it tends to result in a high proportion of credences of users $X_i$ at times $t_i$ for which the corresponding degrees of confidence in the trustworthiness of the AI satisfy^15^

2 $$\begin{aligned} \Big | c_{(t_i,X_i)}[{\varvec{P}}] - r^{{\varvec{P}}}_S(t_i) \Big | \le \varepsilon ,~~\text {where}~~\varepsilon \ge 0. \end{aligned}$$

In eq. (2), $\varepsilon$ is a free parameter that controls this approximation. The smaller the $\varepsilon$, the better the approximation. If $\varepsilon =0$, all credences contributing to the process reliability are vindicated. The necessity to introduce $\varepsilon$ is pragmatic (Tang, 2016): requiring the assessment process to tend to result in a high proportion of *vindicated* credences would be too restrictive, given eq. (1). A remark inspired by formal epistemology: the quantity $\text {L}\left( c_{(t_i,X_i)}[{\varvec{P}}]\right) = 1 -\Big | c_{(t_i,X_i)}[{\varvec{P}}]-r^{{\varvec{P}}}_S(t_i)\Big |$ is the linear score of $X_i$’s credence $c_{(t_i,X_i)}[{\varvec{P}}]$ with objective probability $r^{{\varvec{P}}}_S(t_i)$ at time $t_i$ (Dunn, 2015). The higher the linear score of a credence, the better its approximation of the objective probability at that time. Clearly, many other scoring rules are possible: for instance, $\text {B}\left( c_{(t_i,X_i)}[{\varvec{P}}]\right) =1-\left( c_{(t_i,X_i)}[{\varvec{P}}] - r^{{\varvec{P}}}_S(t_i)\right) ^2$ denotes the Brier score of the credence $c_{(t_i,X_i)}[{\varvec{P}}]$ instead (Pettigrew, 2013).^16^ Finally, our account:

### Definition 3.1

(**Justified credence in the trustworthiness of an AI: a reliabilistic account**) *X*’s credence of $\widehat{\texttt {PTw}}_S(X,t)$ in the trustworthiness of the AI system *S* at time *t* is justified if and only if it is caused by a reliable assessment process ${\mathcal {A}}_S$.

Def. 3.1 states that the justification of our credences in the trustworthiness of an AI is provided by a reliable assessment process, namely, a process that shows the tendency to cause vindicated credence in the trustworthiness of the system. Equivalently, the reliable process shows the tendency to cause perceptions of trustworthiness that are calibrated to the actual levels of trustworthiness of the AI. The justification of these credences relates the perceived and actual levels of trustworthiness of the AI at the time the credences are formed via the objective probability. Our account also suggests that justification comes in degrees. This degree is higher, the higher is the tendency of the assessment process to generate credences that approximate the corresponding objective probabilities. This, in turn, is controlled by the parameter $\epsilon$ in eq. (2).

Keeping things in perspective, our approach is a direct application of Tang’s ‘(Probability)’ account (Tang, 2016). It is reliabilistic, so it is affected by the same limitations of other externalist accounts of justification, which it does not pretend to solve.^17^ Despite this, we believe it has the benefit to clarify how trustworthiness manifests in human-AI interactions and relates to the users’ epistemic attitudes towards it in a rather straightforward way. (Possibly at the cost of introducing an interpretation of the objective probability of AI systems to be trustworthy.) In particular, our notational emphasis on time^18^ descends from this interaction-oriented perspective on trustworthiness: AI systems change over time, so do their trustworthiness levels, users’ roles, behaviors, as well as epistemic attitudes towards technological artefacts. Despite its simplicity, our philosophical, yet interactions-inspired account of justified credences represents a novelty in the literature on justificatory AI. Hopefully, it can pave the way to a more consolidated discourse on the relation between the trustworthiness of an AI, the justification of our epistemic attitudes towards it, and appropriate forms of trusting and reliance in the system. Given the number of different approaches that are currently promoted in the philosophical and computer science literature,^19^ this seems to be a welcomed prospect. Further, it can inspire empirical research in human-AI interactions, as the reliability over time is a measurable property of the assessment process. We will briefly discuss this point in the forthcoming sections.

One final remark before addressing the measurability of the assessment process. Although our account and ‘computational reliabilism’ (CR) (Durán & Jongsma, 2021) both try providing a justification to certain epistemic stances we hold towards AI systems, the two approaches differ in their motivation and approach. First, for the purposes of CR, an AI is de facto identified with its algorithm, i.e., a computational process that generates predictions. In fact, CR provides justification for believing the outputs of (machine learning) algorithms in AI systems (Durán & Jongsma, 2021). Doing so, it ascribes ‘trustworthiness’ to the algorithmic predictions where, arguably, trustworthiness is intended as a synonym of accuracy (Durán & Jongsma, 2021).

In our approach, we consider an AI system as a technical artefact comprising multiple parts that interact with users in different ways over time. This complexity is reflected by what we introduced as the AI’s trustworthiness, which comprises, but it is not identified with, predictive accuracy. Further, we motivated this approach by the need of understanding how the trustworthiness of AI systems manifests over time and relates to agents, their mental processes, and actions in human-AI interactions. CR is introduced as an ‘epistemic manoeuvre’ to deal with the problem of the epistemic opacity of modern machine learning algorithms instead (Humphreys, 2009; Durán & Formanek, 2018; Durán & Jongsma, 2021). Then, we promote credences as the appropriate epistemic attitudes towards the trustworthiness of AI systems, while CR focuses on beliefs as in Goldman’s original account of reliabilism (Goldman, 1979). Finally, we ascribe reliability to the credence-generating process that leads to the user’s perception of the actual trustworthiness of the AI instead to the algorithm that is part of the system, as it is proposed in CR.^20^

### Measuring the Reliability of the Assessment Process of an AI System

Let us fix a time interval $[t_1,t_N]$. We introduce a measure of the (theoretical) reliability of the assessment process ${\mathcal {A}}_{S}$ of an AI system *S* in that time interval. We suppose that *N* agents $X_1,\dots ,X_N$ hold a set ${\mathcal {C}}$ of credences $c_{(t_i,X_i)}\left[ {\varvec{p}}\right]$ in the trustworthiness of the system *S* at times $t_1,\dots ,t_N$. (Similarly, we can suppose that an agent *X* holds *N* credences in the given time interval.) The reliability of the process ${\mathcal {A}}_{S}$ on ${\mathcal {C}}$, given the approximation level $\varepsilon$, is given by

3 $$\begin{aligned} & r^{\text {Q},{\mathcal {A}}_{S}}\left( {\mathcal {C}}|\varepsilon \right) = \frac{1}{1+\varepsilon }\cdot \langle \text {Q}\left( {\mathcal {C}}\right) \rangle , \end{aligned}$$

where $\langle \text {Q}\left( {\mathcal {C}}\right) \rangle := \frac{1}{N}\sum _{i=1}^N \text {Q}\left( c_{(t_i,X_i)}\left[ {\varvec{p}}\right] \right)$ and $\text {Q}\left( c_{(t_i,X_i)}\left[ {\varvec{p}}\right] \right)$ denotes a scoring rule, such as the linear or the Brier score. Equation (3) states that the reliability of ${\mathcal {A}}_{S}$ is the average epistemic utility (Pettigrew, 2013) on ${\mathcal {C}}$ in the time interval $[t_1,t_N]$, rescaled by a factor $f(\varepsilon )=\frac{1}{1+\varepsilon }$. For any $\varepsilon$, $r^{\text {Q},{\mathcal {A}}_{S}}\left( {\mathcal {C}}|\varepsilon \right)$ attains its minimum when either all credences are ‘*beliefs*’^21^ in the trustworthiness of *S* and its objective probability is equal to zero in $[t_1,t_N]$, or all credences are ‘*disbeliefs*’^22^ in the trustworthiness of the system and the objective probability in $[t_1,t_N]$ is equal to one. It reaches its maximum if and only if all credences in $[t_1,t_N]$ are vindicated. In eq. (3), the lower $\varepsilon$, i.e., the better the approximation in eq. (2), the higher $f(\varepsilon )$, and, therefore, the higher the reliability of ${\mathcal {A}}_{S}$, other things equal. In particular, $f(\varepsilon )$ reaches its maximum at $\varepsilon =0$, which corresponds to defining the reliability of ${\mathcal {A}}_{S}$ only in terms of vindicated credences.

### Some Recommendations for Empirical Studies and the Ethics of AI

Finally, we briefly comment on how our account of justified credence in the trustworthiness of AI can inform research in human-AI interactions and the field of AI ethics. The idea is to analyze empirically the relation between the reliability of the assessment process conducted by the users of an AI system and the actual levels of trustworthiness of the AI over time. As discussed in Section “Believing with Degrees in the Trustworthiness of AI”, this is equivalent to study the calibration between the perception of the trustworthiness of an AI and its actual levels. This involves empirically studying how providing users with information about the AI’s design process, retrainings, malfunctions, and past trustworthiness analyses affects their perception of the AI’s trustworthiness.

We posit that this information models the (unknown) objective probability of a system to be trustworthy over time. Researchers can conduct experiments with participants interacting with different versions of the AI, each offering varying details about its history, including documentation on the design of the AI or its audits, time-relative certification labels (Scharowski et al., 2023), and social transparency cues (Ehsan et al., 2021). In addition, experimenters could also simulate different real-world scenarios of AI deployment (e.g., ‘stable AI,’ ‘slowly degrading AI,’ and ‘quickly degrading AI’). By assessing participants’ perceived trustworthiness levels during interactions, researchers can calculate the reliability of the assessment process and examine how variations in trustworthiness levels impact it.

Finally, these studies can investigate the relation between the reliability of the assessment of the trustworthiness of the AI and users’ appropriate reliance on it, measuring their ability to discriminate between correct and incorrect AI’s predictions and act appropriately on them (Schemmer, 2022). To do so, one can manipulate the properties constituting trustworthiness of the system—that the users assess during their interactions with the AI—and assess which manipulation causes (in an interventionist sense) levels of reliability that positively correlate with the correct appraisal of the AI’s capabilities over time. These investigations can advance the discourse on ‘Trustworthy AI’ and, more in general, the field of AI ethics by identifying which AI ‘contracts’ are conducive to the calibration of users’ perceptions of trustworthiness and contribute to the reliability of their assessment process. This, in turn, can help to safeguard user autonomy and protect the public from the misattributions of capabilities and epistemic roles to AI (Ferrario et al., 2024), as well as their misuse and mistrust, promoting societal acceptance and the responsible use of AI technologies.

## Conclusions

Holding justified epistemic attitudes towards the trustworthiness of AI is an important element of the reliance on systems that support our decision-making through their predictions in a myriad of real-world applications. The approach proposed in this work aimed to characterize the trustworthiness of AI systems in a time-relative context, identifying the most appropriate attitudes that capture the different facets of trustworthiness over time and providing an account of justification of these attitudes. As a result, we offer a reliabilistic account of the justification of credence in the trustworthiness of AI systems that allows studying the relation between the reliability of the credence-forming process and the manipulation of the trustworthiness of the AI over time. This, in particular, paves the way to understanding and measuring in empirical studies how to calibrate our perception of the trustworthiness of an AI to the properties that make it worthy of our trust and the relation between the reliability of our assessments and appropriate reliance in the system.

## Data Availability

Not applicable.

## Footnotes

1 In the literature, the terms ‘capability’ (Jacovi et al., 2021), ‘attribute’ (Mattioli et al., 2024), or ‘feature’ (Laux et al., 2024) are used as well. In this work, we will use ‘property’ denoting an inherent characteristic or quality of the AI system that results from its design.

2 The interested reader may consult (Jobin et al., 2019; Floridi et al., 2020; Li et al., 2023; Floridi & Cowls, 2022; Loi et al., 2019; Hagendorff, 2020; Kaur et al., 2022; Díaz-Rodríguez et al., 2023) among others.

3 The life-cycle perspective of AI systems captures the evolution of these machine learning-based artifacts over time (Ng et al., 2022) For instance, in healthcare, the collection of patient data allows the development of machine learning models that serve medical AI systems to predict the goal of care preferences of unconscious patients in the intensive care unit. These systems must be retrained and redeployed to adapt to changes in the target population, the introduction of new medical interventions for patients in intensive care, or the detection of critical issues like bias (Ferrario et al., 2023).

4 The designers’ estimation of this level at (re-)design can be the result of different, quantitative methods. For instance, to measure the safety of an AI, designers may compute the percentage of successfully repelled cyber-attacks during test runs. Accuracy can be measured by computing performance metrics on out-of-sample datasets.

5 The aggregation can be realized mathematically, for instance, as a weighted mean. Weighting allows emphasizing the role of the properties constituting trustworthiness differently. In general, we do not believe there exists a theoretically-motivated choice for this aggregation.

6 https://www.merriam-webster.com/dictionary/assessment.

7 In fact, we are not suggesting that the assessment of the system’s trustworthiness by a user *X* is performed on purely cognitive grounds. We are agnostic to the nature of the mental states leading to the perceived trustworthiness of the system by user *X*. However, we stipulate that, during the assessment, the interaction with the AI supports the formation of a mental model on the system’s trustworthiness.

8 For the sake of clarity, we consider $\widehat{\texttt {PTw}}_S(t,X)$ as a numerical level of perceived trustworthiness. With this we are not suggesting that the result of each user’s assessment is a numerical value but, rather, that a quantification of the perceived trustworthiness is in general possible (e.g., by questionnaires with answers on a numerical scale).

9 For instance, Tang invites to witness the oddity of “saying that a credence of 0.5 in the streets being wet is true or false” (Tang, 2016, p. 4). Note that this is different to say that the content of the credence, i.e., that the streets are wet, is true or false. (Which is a valid statement.)

10 Probabilities are objective if they are not subjective. A probability is subjective if two agents with the same evidence can disagree as to a probability value and yet neither of them be wrong (Williamson, 2009).

11 AI is an epistemic technology designed to predict (Alvarado, 2022b, 2023; Ferrario et al., 2024). The different kinds of AI systems achieve this through distinct implementations that vary in terms of prediction type and format, prediction frequency, data processing methods, and dedicated system interfaces, among others.

12 Objective probabilities of events are known in idealized experiments, such as tossing fair coins or picking a card from a new deck. In these cases, our intuition suggests associating objective probabilities to basic events–like landing heads in a fair coin toss (50%) or picking the king of hearts (1.92%).

13 A technical remark. In our account, the level of actual trustworthiness $\texttt {Tw}_{S}(t)$ of the system *S* at time *t* is used to define the theoretical conditions under which *P* is true at time *t*–specifically, whether $\texttt {Tw}_{S}(t) > a_S$ holds. However, in practice, this condition can only be empirically verified a posteriori, i.e., after *t*, by implementing the designers’ procedures to assess the actual trustworthiness of the system.

14 A reader may propose that beliefs, such as ‘the AI is trustworthy to a degree *x*,’ should be the object of an epistemological analysis of people’s stances towards the trustworthiness of AI systems instead. However, holding these beliefs is hardly tenable from a pragmatic point of view. In fact, it seems difficult to hold them as result of real-world interactions where users may not have the capabilities (and time) to arrive at these precise quantification of the trustworthiness of an AI. (An exception may be designers involved in the testing of an AI before its deployment—still a rather specific type of interaction.) These beliefs are more appropriate for simpler properties of the AI, such as accuracy, whose degrees are measured by standardized procedures.

15 For simplicity, throughout Section “Justifying Credence in the Trustworthiness of AI Systems: A Reliabilistic Account”, we suppose that each agent $X_i$ holds only one credence. Extending this to multiple credences per agent is straightforward, though it makes notation less readable.

16 These scores represent two possible realizations of the ‘epistemic utility’ of $X_i$’s credence of $\widehat{\texttt {PTw}}_S(t_i,X_i)$ in the trustworthiness of the system *S* at time $t_i$, given the objective probability $r^{{\varvec{P}}}_S(t_i)$ (Pettigrew, 2013). Clearly, a credence $c_{(t,X)}[{\varvec{P}}]$ is vindicated if and only if $\text {L}\left( c_{(t,X)}[{\varvec{P}}]\right) =\text {B}\left( c_{(t,X)}[{\varvec{P}}]\right) =1$.

17 That said, classical criticisms of reliabilism, such as ‘the generality problem,’ have been addressed by numerous scholars over time-see, for instance (Comesaña, 2006; Beebe, 2004). Additionally, the fact that users’ assessments of the trustworthiness of an AI system are based on a limited number of interactions, by design of these systems and their interfaces, provides a degree of protection against deviations from ‘normal worlds’ (Goldman, 1986), which is at the basis of Goldman’s answer to Bonjour ‘clairvoyance’ criticism (BonJour, 1980). This helps reduce the number of potentially reliable process types, which directly addresses the core of the generality problem.

18 Some readers may find this emphasis unnecessary, or, at least of secondary importance with respect to a point-wise-in-time discussion of the different facets of trustworthiness of an AI. That said, the type of interactions and, a fortiori, the different epistemic attitudes that human agents may have towards an AI depend on the current life cycle step of the system. Therefore, a time-relative description of trustworthiness and credences in it allowed us considering all these different aspects in a compact way. Finally, we note that other authors have emphasized the importance of time for reliabilist accounts of justificatory AI as well (Buijsman, 2024).

19 A glance at works, such as Jacovi et al. (2021); Durán and Jongsma (2021); Alvarado (2022b, 2023); Ferrario and Loi (2022); Freiman (2023); Nickel (2022); Loi et al. (2023), should give a sense of some of these approaches.

20 Finally, we note that, although CR is a widely discussed account of justification in relation to AI systems (Durán & Jongsma, 2021; Ferrario & Loi, 2022; Nickel, 2022) it does not encounter the favor of all authors. In fact, considering deep learning methods supporting medical diagnoses, Alvarado argues that the reliability of these methods has not yet been established and, therefore, cannot be used to provide justification to beliefs (Alvarado, 2022a, 2024).

21 That is, the degree of confidence $\widehat{\texttt {PTw}}_S(t_i,X_i)$ is equal to one for all credences in ${\mathcal {C}}$.

22 That is, the degree of confidence $\widehat{\texttt {PTw}}_S(t_i,X_i)$ is equal to zero for all credences in ${\mathcal {C}}$.
